# A cluster-randomised controlled trial comparing school and community-based deworming for soil transmitted helminth control in school-age children: the CoDe-STH trial protocol

**DOI:** 10.1186/s12879-019-4449-6

**Published:** 2019-09-18

**Authors:** Naomi E. Clarke, Dinh Ng-Nguyen, Rebecca J. Traub, Archie C. A. Clements, Kate Halton, Roy M. Anderson, Darren J. Gray, Luc E. Coffeng, John M. Kaldor, Susana Vaz Nery

**Affiliations:** 1 The Kirby Institute for Infection and Immunity in Society, University of New South Wales, Sydney, Australia; 2 Faculty of Animal Sciences and Veterinary Medicine, Tay Nguyen University, Dak Lak, Vietnam; 3 Faculty of Veterinary and Agricultural Sciences, University of Melbourne, Parkville, Australia; 4 Faculty of Health Sciences, Curtin University, Perth, Australia; 5 Telethon Kids Institute, Perth, Australia; 6 Institute of Health and Biomedical Innovation, Queensland University of Technology, Brisbane, Australia; 7 Department of Infectious Disease Epidemiology, School of Public Health, Faculty of Medicine, Imperial College London, London, UK; 8 Research School of Population Health, Australian National University, Canberra, Australia; 9 Department of Public Health, Erasmus MC, University Medical Center Rotterdam, Rotterdam, The Netherlands

**Keywords:** Soil-transmitted helminths, Hookworm, Mass drug administration, Albendazole

## Abstract

**Background:**

Current guidelines and targets for soil-transmitted helminth (STH) control focus on school-based deworming for school-age children, given the high risk of associated morbidity in this age group. However, expanding deworming to all age groups may achieve improved STH control among both the community in general and school-age children, by reducing their risk of reinfection. This trial aims to compare school-based targeted deworming with community-wide mass deworming in terms of impact on STH infections among school-age children.

**Methods:**

The CoDe-STH (Community Deworming against STH) trial is a cluster-randomised controlled trial (RCT) in 64 primary schools in Dak Lak province, Vietnam. The control arm will receive one round of school-based targeted deworming with albendazole, while in the intervention arm, community-wide mass deworming with albendazole will be implemented alongside school-based deworming. Prevalence of STH infections will be measured in school-age children at baseline and 12 months following deworming. The primary outcome is hookworm prevalence in school-age children at 12 months, by quantitative PCR. Analysis will be intention-to-treat, with outcomes compared between study arms using generalised linear and non-linear mixed models. Additionally, cost-effectiveness of mass and targeted deworming will be calculated and compared, and focus group discussions and interviews will be used to assess acceptability and feasibility of deworming approaches. Individual based stochastic models will be used to predict the impact of mass and targeted deworming strategies beyond the RCT timeframe to assess the likelihood of parasite population ‘bounce-back’ if deworming is ceased due to low STH prevalence.

**Discussion:**

The first large-scale trial comparing mass and targeted deworming for STH control in South East Asia will provide key information for policy makers regarding the optimal design of STH control programs.

**Trial registration:**

ACTRN12619000309189.

## Background

Soil-transmitted helminths (STH), comprising roundworm (*Ascaris lumbricoides*), hookworms (*Necator americanus*, *Anyclostoma duodenale* and *Ancylostoma ceylanicum*), and whipworm (*Trichuris trichiura*) are the most prevalent of the neglected tropical diseases (NTDs), a group of illnesses that primarily affect people in low income settings, contributing to an ongoing poverty cycle [1]. Morbidity associated with STH infections includes impaired physical and cognitive development and iron-deficiency anaemia, with children and women of reproductive age at particular risk [2–4].

Over the past decade a global commitment to improving NTD control has seen the scale-up of STH control programs in endemic areas [5], predominantly based on large-scale preventive chemotherapy, in which deworming drugs—mebendazole or albendazole—are delivered at regular intervals [5]. Such programs can be either “targeted” to specific at-risk groups (e.g., by age group or occupation), or “mass”, with treatment delivered to the entire population [6].

The World Health Organization (WHO) 2020 target for STH control is regular deworming of 75% of preschool- and school-age children (aged 2–4 years and 5–12 years, respectively) in endemic areas [7]. In 2017, 596 million school-age children and 227 million preschool-age children required preventive chemotherapy for STH [8]. The success of deworming programs has been bolstered by the donation of 600 million annual doses of mebendazole and albendazole by pharmaceutical companies (Johnson & Johnson and Glaxo Smith Kline) to cover school-age children [9], with recent figures suggesting that overall progress is on track to meet the 2020 targets [8]. School-based targeted deworming programs represent the mainstay of STH control efforts, utilising existing school-based infrastructure to reach a large proportion of school-age children, with teachers acting as drug distributors [6, 10, 11].

WHO guidelines published in 2017 recommend regular deworming of not only preschool- and school-age children, but also children aged 12–23 months, adolescent girls (aged 10–19 years) and women of childbearing age (15–49 years) in endemic areas [12], and there have been calls for expansion to treat all age groups [13]. Community-wide treatment is successfully used for other NTDs, including lymphatic filariasis, trachoma, and onchocerciasis [14–16]. Mathematical modelling has demonstrated that deworming of school-age children is unlikely to have a significant impact on community-wide transmission, especially for hookworm, because large reservoirs of infection exist in adults [17–19]. Furthermore, a systematic review and meta-analysis of studies conducting either mass or targeted deworming showed that treating entire communities significantly enhances STH prevalence reduction among school-age children [20].

Our recent pilot study in Timor-Leste compared school- and community-based approaches to STH control, providing proof of principle for the hypothesis that a community-wide approach leads to greater STH prevalence reduction among school-age children [21]. The recent TUMIKIA study in Kenya reported lower hookworm prevalence across all age groups following annual community-wide deworming, compared to school-based deworming [22]. The ongoing Deworm3 study in Benin, Malawi and India is also comparing community-wide and school-based deworming [23]. No community trials comparing mass and targeted deworming have been conducted in South East Asia.

The CoDe-STH trial aims to test the hypothesis that community-wide STH control programs will lead to fewer reinfections among school-age children in a South East Asian setting. Specific objectives are:

1. To compare the impact of targeted deworming and mass deworming on the prevalence and intensity of STH infections among school-age children;
2. To evaluate and compare the cost-effectiveness of targeted deworming and mass deworming;
3. To examine the acceptability and feasibility of school-based and community-based deworming;
4. To predict the long-term impact of targeted deworming and mass deworming on STH transmission using individual based stochastic models of parasite transmission.

## Methods/design

This study protocol has been prepared according to the SPIRIT (Standard Protocol Items: Recommendations for Intervention Trials) recommendations [24].

### Overview of study design

**Fig. 1:**
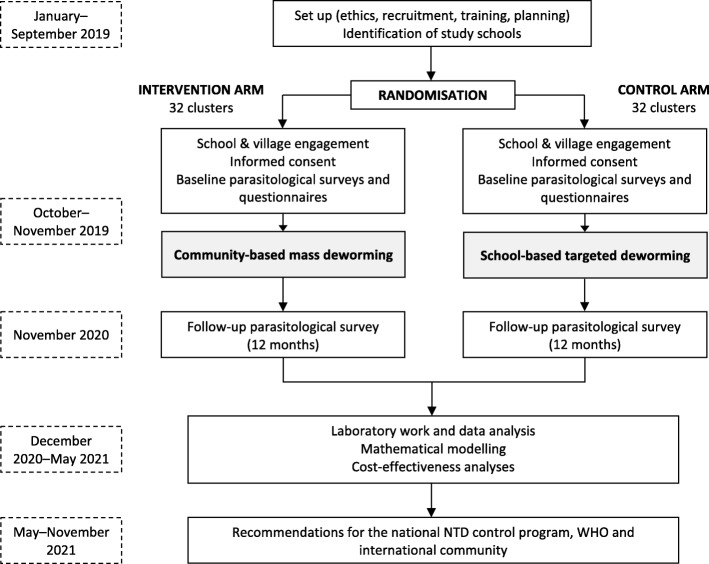
Flow diagram of the trial

The CoDe-STH trial design is depicted in Fig. 1. The trial is a two-arm cluster randomised controlled trial (RCT), with each cluster being a primary school located in Dak Lak Province, Vietnam. It has been designed to evaluate the impact of community-wide mass deworming on STH infections among school-age children, compared to primary school-based targeted deworming. In the control arm (32 clusters), one round of school-based targeted deworming with albendazole will be conducted at primary schools. In the intervention arm (32 clusters), community-based mass deworming with albendazole will be implemented alongside school-based deworming. STH infections will be detected using quantitative polymerase chain reaction (qPCR) on stool samples collected from school-age children during surveys at baseline and 12 months following deworming. A 12-month follow-up period was selected based on the Vietnamese national deworming schedule, as well as evidence that one round of deworming will lead to significant reduction in STH prevalence at 12 months [20].

This trial has received ethical approval from the Human Research Ethics Committees at the University of New South Wales (HC190136) and Tay Nguyen University (1804-QĐ-ĐHTN-TCCB), and is registered with the Australian New Zealand Clinical Trials Registry (registration number ACTRN12619000309189).

### Study setting

**Fig. 2:**
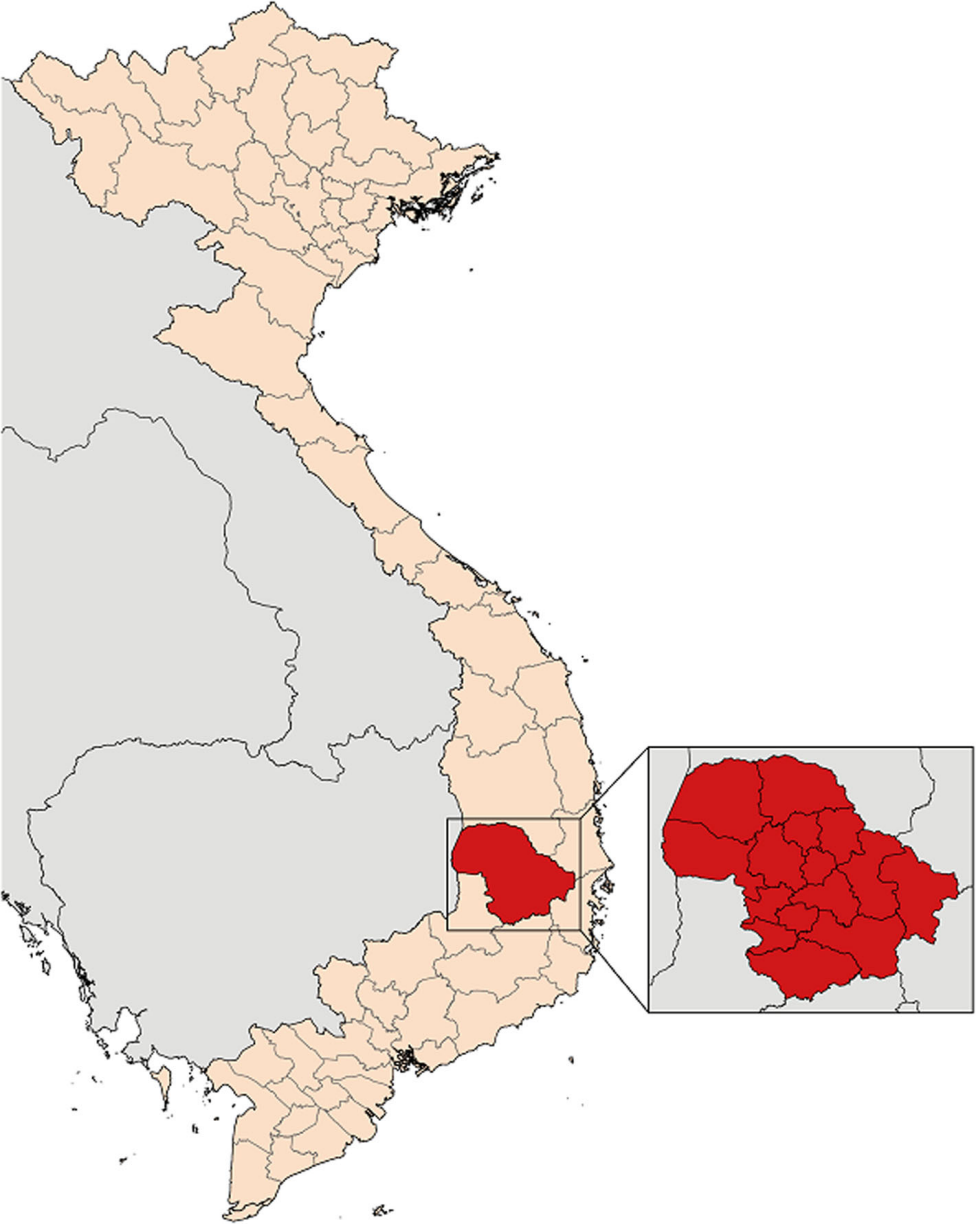
Map of Vietnam showing Dak Lak province and its second-tier administrative units (districts, town and city)

Dak Lak province is located in the Central Highlands of Vietnam (see Fig. 2), and has a population of approximately 1.9 million [25]. The province is comprised of one provincial city (Buôn Ma Thuột), one district-level town (Buôn Hồ), and 13 districts. Together, the provincial city, district-level town, and districts are comprised of 182 communes, wards and towns, with each further subdivided into hamlets, generally comprising between 100 and 1000 households. The trial will take place in all 13 districts.

Dak Lak province has implemented large-scale deworming of primary school-age children with mebendazole or albendazole since 2007 as part of the national STH control program. Deworming has taken place twice annually since 2014, apart from 2018 because of difficulties in procuring mebendazole. Due to decreasing STH prevalence, from 2019 onwards, deworming will be conducted once annually (D. Nguyen, personal communication). We conducted a preliminary survey in 13 primary schools across Dak Lak province in December 2018 in preparation for the CoDe-STH trial. This survey revealed an overall hookworm prevalence, using qPCR, of 20.6%, with school-level prevalences ranging from 3.2 to 55.2% (unpublished data). Prevalence of *A. lumbricoides* and *T. trichiura* were much lower at 2.5 and 1.1%, respectively. These findings were very similar to those reported in a survey of four primary schools conducted in 2015 [26].

The research field team will consist of a local project manager and six field supervisors from Tay Nguyen University, four local staff recruited from the Department of Health and Department of Education in each district, and local hamlet health workers (one per participating hamlet).

### Intervention

Albendazole will be used as the deworming drug for the study intervention, given that it has higher efficacy than mebendazole against hookworm [27] and is more widely used in STH control programs [5]. One round of deworming will be conducted in both study arms in November 2019. In the control arm, we will conduct school-based deworming only. All children attending each study school (typically aged 6-11 years) will receive the recommended 400 mg dose of albendazole [12], using the same procedures as the regular Ministry of Health deworming program.

In the intervention arm, we will conduct both school-based and community-wide deworming. All schoolchildren will receive albendazole as described above. In addition, community-based deworming will be implemented in hamlets where children enrolled in the primary school live. For each school, we will select enough hamlets for community deworming to ensure that at least 70% of children enrolled in the school are resident in a treated hamlet. While 100% coverage of hamlets in catchment communities would have been ideal, the lower proportion was selected due to financial and logistical constraints. Preliminary data from the Department of Education and Training indicate that 70% coverage will generally require treating between 1 and 4 hamlets per school. In each selected hamlet, house-by-house visits will be conducted and albendazole 400 mg offered to every household member, excluding children who already received albendazole at school. As per WHO guidelines, children under the age of 1 year will not receive albendazole, and children aged 12–23 months will receive 200 mg [12]. Pregnant women in the first trimester (defined as all those who report being pregnant but have not felt the baby move, and those who report missing their last menstrual period) will also be excluded from treatment following WHO guidelines.

In both arms, albendazole will be distributed by hamlet health workers and district-level Department of Health staff. All doses will be taken under direct observation. Due to the nature of the intervention, neither study participants nor research team members can be blinded.

Albendazole is known to be extremely safe when delivered in single doses [28], with hundreds of millions of doses delivered annually in deworming programs for STH and lymphatic filariasis [8, 15]. Participants will be advised to attend the local commune medical station if they are unwell following drug distribution. Medical stations will be advised of the deworming activities. Passive monitoring of adverse events will be undertaken as described further below.

### Study outcomes

The primary outcome is the prevalence of hookworm infection (unspeciated) detected using qPCR, 12 months after deworming. Secondary outcomes are prevalence of *N. americanus*, *A. duodenale*, *A. ceylanicum* (i.e. species-specific hookworm infections), *Ascaris* spp., and *T. trichiura* by qPCR; mean intensity of hookworm infection, calculated as the average number of eggs per gram of faeces; treatment coverage with albendazole (i.e., the proportion of eligible schoolchildren and community members who receive albendazole); and adverse events following albendazole distribution.

### Selection and randomisation of clusters

Schools are eligible to participate in the study if they meet the following criteria:

- Defined by the Department of Education and Training as a primary school (grades 1–5)
- Have between 200 and 450 students enrolled (due to sample size requirements and operational constraints)
- Located in a rural, remote, or very remote area, as defined by Department of Education and Training.

In June 2019, a list of schools meeting these criteria was generated with assistance from the Department of Education and Training. Out of 423 primary schools in Dak Lak province, 129 met eligibility criteria, and 64 were randomly selected from this list, stratified by district to ensure good geographic coverage. To ensure that no study schools shared hamlets in their catchment areas, no more than one school was selected from a given commune. The remaining 65 eligible schools were retained as potential replacement schools.

Selected study schools and replacement schools were randomised 1:1 to the control and intervention arms, stratified by district. Randomisation was performed by an independent, blinded statistician, using a computerised random number generator.

### Participants

Participation in data collection involves providing a stool sample and completing a questionnaire. For each school, participants in data collection will be school-age children in grades 1–4 (typically aged 6–10), residing in hamlets that collectively include as residents at least 70% of children enrolled in the primary school. The participants will be followed across both data collection time points; therefore, children in grade 5 will not be recruited for data collection, because they will no longer be in primary school at the 12-month follow-up. All children in grades 1–4 who reside in the selected hamlets will be eligible to participate in the study, provided their parents provide informed consent (see below). In both arms, in accordance with national and WHO deworming policies, all children attending school will receive albendazole, regardless of whether they participate in data collection.

### Engagement and recruitment

Key stakeholders in Dak Lak province, including the Center for Parasitic Disease Control and Prevention and the Department of Education and Training, have been involved in this study from an early stage, and have provided input into the study design, timing, and procedures.

In the several months prior to baseline study visits, initial engagement visits will be conducted in all 13 districts of Dak Lak province. During these visits, members of the research team will hold meetings with the district level Department of Health and Department of Education and Training, to describe the study aims, interventions, and data collection procedures. Each selected study school will then be visited and the study described to the school principals and teachers. Verbal consent will be sought from the principal to conduct data collection and deworming in the school. If consent is not obtained (a circumstance considered unlikely by local collaborators), a replacement school will be randomly selected from the pre-generated, pre-randomised replacement list of schools. Once a principal has agreed, a meeting will be held with commune and hamlet leaders from participating hamlets, to describe the study, seek their support in encouraging parent and community participation, and identify a health worker per hamlet to assist with community mobilisation. For hamlets in the intervention arm, verbal consent will be sought from the stakeholders above to conduct community deworming.

At study baseline, each school principal will organise a meeting at the school between the research team and parents/guardians of children who live in the selected hamlets. Hamlet health workers will encourage parent attendance. The trial purpose, procedures, data collection, and interventions will be explained to parents, and an opportunity provided for questions. Written information sheets will be distributed (see Additional file 1 for the English version) and written informed consent will be sought from parents/guardians by members of the research team for children to participate in data collection. For parents who do not attend the meeting, written information statements and consent forms will be sent home. Consent will not be sought for children to receive albendazole at school, because school-based deworming is usual practice in Vietnam, and the trial will follow similar procedures to those used by the Ministry of Health deworming program.

In intervention clusters, hamlet-level meetings will be arranged by the hamlet leader, at which the research team will explain the study to members of communities that will be offered the deworming intervention. Written informed consent will subsequently be sought by members of the research team during house-to-house visits for albendazole distribution. Similar procedures will be used for participant engagement and recruitment at follow-up.

### Data collection

Once written information consent has been obtained from parents/guardians, the study will be explained to the children from hamlets selected for participation in data collection. A questionnaire (see Additional file 2 for the English version) will be administered at study baseline to each child and his/her parent or guardian. This will be done at the primary school immediately following the parent meeting, via interview with a trained member of the research team. The interview will be conducted in Vietnamese and will take approximately 10 min to complete. Questions will relate to participants’ defecation and hygiene practices (asked to children), as well as household water sources, home environment and socioeconomic status (asked to parents).

Parasitological surveys will be done at study baseline and 12 months after deworming. On the first day of data collection, participating children will be instructed on how to collect a stool sample and provided with a collection kit. They will be asked to collect an early morning stool sample the following morning and bring it with them to school. The research team will be present at the school for 3 days in total, so will be present to receive stool samples for 2 days. Research team members will process stool samples immediately in the field, preserving a 3-g aliquot in 5% (w/v) potassium dichromate. Aliquots will be kept chilled until refrigerated.

Preserved stool samples will be sent to the University of Melbourne, Australia, for analysis using quantitative PCR [29, 30]. Following washing and beating steps, DNA extraction will be performed using the Maxwell® RSC PureFood GMO and Authentication Kit on a Maxwell® RSC 48 Instrument (Promega Corporation). This will be followed by two real-time multiplex PCR assays: one to detect and quantify *N. americanus*, *A. duodenale*, and *A. ceylanicum*, and the other to detect and quantify *Ascaris* spp. and *T. trichiura*, using Equine Herpes Virus 4 and human DNA as qPCR internal and extraction controls, respectively. Infection intensity will be determined using standardised curves of faeces seeded with eggs of each species, to convert cycle threshold (Ct) values obtained from PCR into eggs per gram of faeces. Laboratory staff conducting the STH diagnosis will be blinded to participants’ allocation.

Treatment coverage will be documented by recording the number of albendazole doses delivered and taken by students at each school, as well as the total number of students enrolled in each school. In the intervention arm, the number of albendazole doses delivered and taken by each household will be documented, and the population of each hamlet obtained from the hamlet leader.

Adverse events will be recorded using passive monitoring. At the time of deworming, teachers and hamlet health workers will be trained by the research team to recognise and document adverse events related to deworming, including abdominal pain, diarrhoea, nausea, headache and dizziness [28]. Members of the research team will return to study schools 1 week following deworming, to collate information about potential adverse events from teachers and hamlet health workers, as well as from commune medical stations.

### Sample size calculation

Sample size calculations were based on the primary outcome, prevalence of hookworm infection. To estimate the number of clusters required, we used our previously published generalised linear model to predict the impact of mass and targeted deworming on prevalence reduction in school-age children [20]. Assuming a baseline hookworm prevalence of 20%, the model predicts a relative prevalence reduction of 79% after mass deworming and 45% after targeted deworming, measured 12 months after one round of deworming. Assuming an average cluster size of 120 children in the target age group and an intra-cluster correlation coefficient of 0.12 [31], with a power of 80% and α = 0.05, the sample size required to detect this reduction or greater as significant is 64 primary schools, randomised 1:1 between the two study arms. Given the predicted low starting prevalences of *A. lumbricoides* and *T. trichiura*, the study is not powered to detect a difference between study arms for these STH.

To achieve the required sample size of 120 participants per cluster, we will initially aim to recruit at least 180 participants per cluster. This will allow for a loss to follow-up of up to 30%. The estimated total number of participants in data collection is 7680.

### Data management

Data will be collected using electronic tablets, with REDCap electronic data capture tools [32] hosted at UNSW Sydney. Data will be collected offline and sent to the secure REDCap server daily using a secure wireless connection. Results of the qPCR analysis will be entered into a customised database and sent securely to UNSW Sydney. Regular data management meetings will be held to examine data quality and completeness, and review any queries. Trial data will be accessible only by a limited number of study investigators. De-identified data will also be provided to the Center for Parasitic Disease Control & Prevention in Dak Lak province.

### Statistical analysis

Intention-to-treat analyses will be conducted to compare study outcomes between the intervention and control arms. Unadjusted analyses will involve comparing infection prevalence and intensity between study arms at study follow-up, using univariable regression analyses accounting for clustering by school. For adjusted analyses, generalised linear and non-linear mixed models will be used to account for within and between cluster variability. For infection prevalence, the outcome will be the infection status of the individual, so Bernoulli logistic regression will be used. For infection intensity, linear regression models will be used. For all models, age and sex will be entered as covariates (fixed effects), and school as a random effect. The intervention will be entered as a binary fixed effect to estimate differences in prevalence and intensity, and a relative risk of infection, comparing the two arms at each follow-up survey. All analyses will be conducted using a 5% level of significance.

Baseline characteristics will be compared between the intervention and control arms using descriptive statistics. If there is a need to adjust for differences in the baseline characteristics of the intervention and control groups, additional analyses will be conducted incorporating variables measured in the study questionnaires as covariates, allowing an adjusted effect estimate to be calculated for the intervention.

### Assessment of acceptability and feasibility

A mixed-methods study will be undertaken to identify perceptions, barriers and facilitators to community-wide deworming in Vietnam, in order to inform policymakers regarding implementation and scale-up of community-based deworming. The acceptability of school-based and community-wide deworming will be examined using a series of focus group discussions (FGDs) with community members, school headmasters, and teachers, conducted following the first round of deworming. The FGDs will take place in randomly selected schools and hamlets in both study arms, stratified by district. Purposive sampling of community members will be used, to ensure inclusion of community members across different age groups (including school-age children), socioeconomic positions, and cultural groups, and representation of both males and females. Individual semi-structured interviews will additionally be held with community leaders, school headmasters, and teachers. FGDs and interviews will explore perceptions of community-based deworming for STH control, and perceived barriers and facilitators at individual and community levels.

Feasibility of community-based deworming for STH control will be explored using individual semi-structured interviews with key stakeholders, to identify perceptions and perceived barriers and facilitators at programmatic and policy levels. Interviews will be held with national, provincial, and district-level staff of the national NTD control program, and health staff at district and commune levels.

Written informed consent will be obtained prior to FGDs and interviews, which will be conducted in Vietnamese and recorded digitally. Following completion of the FGDs and interviews, recordings will be transcribed and translated. Coding and analysis will be done using NVivo software (QSR International, Doncaster, Australia). Key themes will be identified and analysed using thematic analysis.

### Cost-effectiveness analyses

Two analyses of the incremental cost-effectiveness of the community-based strategy compared with the school-based strategy will be undertaken. The first analysis will use a short time frame (12 months) and will be based on cost and health outcomes observed in the trial. Costs will be estimated from the perspective of the Departments of Health and Education, and will include financial expenditure and the opportunity cost of using existing staff and resources. Resource use data will be collected prospectively through project and accounting records, and valued using the international drug price indicator guide, and local market prices and labour costs. Total implementation costs will be organised into consumables versus staffing costs to allow us to estimate the relative contribution of different components to total program costs. The second analysis will use a 10-year time frame to look at the longer-term efficiency of a community-based strategy. Costs for years 2–10 will be extrapolated from expenditure estimates, excluding one off start-up costs. Total annual costs for each control strategy will be reported in Vietnamese Dong (VND) and adjusted to a common cost year, with standard discount rates applied where necessary. Health outcomes will be estimated using the WORMSIM mathematical model to capture the transmission dynamics of hookworm infection [19]. The primary outcome will be Disability-Adjusted Life Years (DALYs) averted due to preventing hookworm infections. This will be calculated using the methods developed for the Global Burden of Disease study [33]. The relative change in costs and effectiveness will be used to calculate an incremental cost-effectiveness ratio for the community-wide and school-based STH control strategies. Results will be presented as both the incremental cost per infection avoided and the incremental cost per DALY averted, to enable comparison with the efficiency of other large scale public health programs. We will undertake one-way and probabilistic sensitivity analyses to understand the robustness of our conclusions.

### Mathematical modelling

Two mathematical models will be used to enhance understanding of trial findings and predict long-term impact of the two deworming approaches. We will use the existing individual-based mathematical model WORMSIM and an individual-based version of that employed by the Imperial College team to simulate qPCR results [19, 34]. Model parameters for the overall transmission rate and inter-individual variation in exposure will be calibrated against baseline distribution of infection intensity and prevalence by age. We will then use the model to predict the impact of interventions in each trial arm given information on deworming coverage and individual treatment compliance, and compare predictions to trial results. To capture and carry forward stochastic and parametric uncertainty, model parameters will be calibrated within a Bayesian framework, using a sequential Monte Carlo approach [35]. Important discrepancies between observed data and model predictions will be explored using alternative plausible model parameterisations in an iterative fashion. Using the model, we will further evaluate how sensitive the achievement of disease control (defined as prevalence of moderate-to-heavy intensity infection < 1%) is to pre-control transmission parameters, including rate of transmission and the uptake of deworming. Finally, we will predict at what overall prevalence level by qPCR stopping mass or targeted deworming will result in transmission being interrupted with a defined probability [36, 37].

### Dissemination

The trial results will be formally presented to the Vietnamese Ministry of Health and the Dak Lak Province Centre for Parasitic Diseases Control and Prevention, and a summary of the results will be provided to the Department of Health in each district of Dak Lak province. Results will also be disseminated to key stakeholders in deworming policy, including the WHO.

The results of the trial will be published in peer-reviewed journals and presented at international conferences. The primary outcomes of the trial will be reported following CONSORT guidelines for cluster RCTs. Results of cost-effectiveness analyses, feasibility and acceptability of school and community deworming, and mathematical modelling will be prepared as separate publications. De-identified data will be available on request, following completion of the trial and publication of the trial results.

## Discussion

The past decade has seen many countries scale up school-based deworming programs to improve STH control, in accordance with current WHO targets that are focused on achieving 75% worldwide coverage of at-risk school- and preschool-age children by 2020 [7]. With under a year remaining to this target, there is vigorous discussion underway regarding STH control targets and recommendations, including for the WHO 2020–2030 NTD Road Map. On the basis of evidence from mathematical and economic modelling studies and meta-analysis, there have been calls to expand deworming programs for STH control community-wide [13, 20, 38].

Empirical evidence from field-based research is needed to confirm the predicted additional benefits of community-wide deworming. The CoDe-STH trial is one of the first RCTs comparing the impact of community-wide mass deworming and school-based targeted deworming on STH infections, and the first in South East Asia. This trial will provide robust evidence regarding the differential impact of school-based and community-wide deworming on STH infections among school-age children, who are at high risk of STH-associated morbidity. The use of qPCR to diagnose and quantify infections among the study population will not only allow for a more accurate determination of disease prevalence and intensity before and after deworming [30, 39], but will also contribute to the evidence base for using more sensitive diagnostic techniques to monitor the impact of deworming. This is an increasingly important consideration as global deworming efforts continue and STH prevalence and intensity declines to that undetectable by microscopy in many regions [37, 40].

Mathematical modelling approaches will be used to determine the longer-term impact of mass and targeted deworming beyond the trial follow-up period, and examine at what true prevalence of infection (determined by the most sensitive diagnostic method) deworming programs can cease, with a high probability that transmission has been interrupted. Furthermore, the CoDe-STH study will include assessment of the cost-effectiveness of mass and targeted deworming, and the acceptability and feasibility of the two approaches within communities, schools, local program implementers, and policymakers. Taken together, the findings from these multiple components of the CoDe-STH trial will provide a comprehensive picture of evidence to policymakers, donors, and other key stakeholders in the NTD sector. This evidence has the potential to inform future WHO guidelines and recommendations for STH control programs, in order to sustain and build on the success of recent deworming efforts, and maximise benefits to at-risk populations worldwide.

## Supplementary information

**Additional file 1.** Parent/guardian information statement.

**Additional file 2.** Study questionnaire.

## Data Availability

Not applicable

## Abbreviations

CoDe-STH: Community Deworming against soil-transmitted helminths
DALY: Disability-adjusted life year
FGD: Focus group discussion
NTD: Neglected tropical diseases
qPCR: quantitative polymerase chain reaction
RCT: Randomised controlled trial
STH: Soil-transmitted helminths
WHO: World Health Organization
