# Supplementary material for: A cluster-randomised controlled trial comparing school and community-based deworming for soil transmitted helminth control in school-age children: the CoDe-STH trial protocol
Source: BMC Infect Dis. 2019 Sep 18;19:822. doi: 10.1186/s12879-019-4449-6 (PMC6751595; doi:10.1186/s12879-019-4449-6)
Supplement: Supplementary file 1 — Additional file 1. Parent/guardian information statement. [file 12879_2019_4449_MOESM1_ESM.pdf]

PARTICIPANT INFORMATION STATEMENT AND CONSENT FORM – Parent Guardian
School and community deworming to control intestinal worm infections
Chief Investigator: Associate Professor Susana Vaz Nery

1. What is the research study about?

This is an invitation for you and your child to take part in a research project looking at how best to control intestinal worm infections. You and your child have been invited to participate because your child attends ________________________ primary school.

Children in Vietnam, and many other countries, receive regular medication to treat intestinal worms at school. In Dak Lak, this is now being done once a year. Regular treatment is given because these infections can come back after treatment. We want to find out if we can reduce intestinal worm infections in children more by treating all members of the community, instead of only treating children.

This study is being conducted in 64 primary schools in Dak Lak province. In 32 of these schools (the "control arm"), deworming medication (albendazole) will be given to primary school children only. This is the usual practice in Vietnam. In the other 32 schools (the "intervention arm"), albendazole will also be given to all community members, in 2 or 3 hamlets that send children to the school. We will do this by visiting all households in the hamlet. Schools will be randomly assigned to either the intervention or control arm of the study.

2. Who is conducting this research?

The principal investigator of this study is Associate Professor Susana Vaz Nery, University of New South Wales, Australia. Other researchers involved in this project are Dr Dinh Nguyen (Tay Nguyen University) and Dr Naomi Clarke (UNSW).

Research funder: This research is being funded by the National Health & Medical Research Council.

3. Inclusion/Exclusion Criteria

All children in grades 1 to 4 (and their parents/guardians) are eligible to participate in this research project. We have selected a sample of children in grades 1 to 4 to take part in this research project.

4. Do my child and I have to take part in this research study?

Participation in this research study is voluntary. If you do not want you or your child to take part, then you do not have to. Your child will still receive regular treatment for intestinal worms whether or not he/she takes part in the research study.

If you decide that you want you and your child to take part in the research study, you will be asked to:

- Read this information statement carefully (ask questions if necessary);
- Sign and return the consent form if you decide to participate in the study;
- Take a copy of this form with you to keep.

5. What does participation in this research require, and are there any risks involved?

If you consent to take part in the research study, this will involve two things:

1. Your child will be asked to provide a sample of their stool. This will happen twice: now and in 12 months' time. At each time, your child will be provided with a container for collecting their stool sample and will be taught how to safely collect the sample. We will ask your child to bring their sample back to the research team at their school. The stool sample will be analysed at the University of Melbourne in Australia, to look for intestinal worm infections.
2. You and your child will be asked to complete a questionnaire. The questionnaire will ask you and your child questions about their access to toilets, drinking water, and your child's hygiene behaviour. It will be done as a confidential interview with a member of the study team, and should

take approximately 10 minutes to complete. Your participation in this part of the project is voluntary. You are not obliged to fill in the questionnaire if you do not wish to.

All children at your child's school will receive deworming medication (albendazole), whether they participate in the study or not. This is done as part of usual health activities in Vietnam. Side effects are uncommon and mild (including stomach pain, nausea, headache, and dizziness), and go away quickly.

There is a small risk when collecting a stool sample that your child may be exposed to infectious diseases. We will teach your child how to collect their stool sample safely and hygienically and will provide gloves.

Some children may feel embarrassed or anxious about being asked to provide a stool sample, or about answering questions about toilets and hygiene. Your child does not have to take part in this study if he/she feels embarrassed or anxious. Even if you agree for your child to take part in the study, he/she does not have to provide a stool sample or answer the questionnaire if he/she decides that he/she does not want to. If you or your child experiences distress because of participating in this study you can tell the research team and they will assist.

6. What are the possible benefits to participation?

This research study will help us to learn about the best way to control intestinal worm infections. We hope that what we learn from this study will help us to improve the way we treat intestinal worm infections in many countries around the world. Your child will continue to receive regular treatment for intestinal worms at school.

7. What will happen to information about my child?

By signing the consent form you consent to the research team collecting and using information about you and your child for the research study. The stool samples will be kept in a secure location in the laboratory, and will be destroyed after six months. DNA extracted from the stool samples will be destroyed after five years. This DNA relates to worms and contains no genetic information about your child. We will keep the information about intestinal worm infections and the information from the questionnaires for five years. We will store this information in an identifiable format in a password protected computer database but the identifying codes will be stored separately, also in a password protected database. Only members of the research team will have access to this.

Researchers at UNSW must store their aggregated data in the UNSW data repository, called ResData. Once the aggregated data is deposited into this repository it will be retained in this system permanently. It will, however, be retained in a format where you and your child's identity will not be known.

The information you provide is personal information for the purposes of the Privacy and Personal Information Protection Act 1998 (NSW). You have the right of access to personal information held about you by the University, the right to request correction and amendment of it, and the right to make a complaint about a breach of the Information Protection Principles as contained in the PPIP Act. Further information on how the University protects personal information is available in the **UNSW Privacy Management Plan**.

8. How and when will I find out what the results of the research study are?

The research team will publish and report the results of the research study in a variety of ways. All information published will be done in a way that will not identify you or your child.

The results of the research study will be provided to your child's school and the district Centre for Public Health at the end of the study. If you would like to receive a copy of the results you can tell your child's head teacher or the Centre for Public Health at your district.

9. What if I want to withdraw my child from the research study?

Even if you consent for your child to participate, he/she does not have to provide a stool sample or answer the questionnaire. You and/or your child can decide at any time that you do not want to continue participating in the research project. If you decide that you no longer want you and your child to participate in the research project, you may fill in the "Withdrawal of consent" form at the end of this document, or you may contact the research team and inform them. Any information about you and your child will then be removed from the research project.

Your decision not to participate or to withdraw from the study will not affect your relationship with your child's teachers, school, local health authorities, the Centre for Parasitic Diseases Control & Prevention, Tay Nguyen University, or the University of New South Wales. Your child will still receive treatment for intestinal worms as part of the usual national control program.

10. What should I do if I have questions about my child's involvement in the research study?

If you want any further information about this project before you sign the consent form, you can ask the research team. If you have any problems which may be related to your involvement in the project, you can contact the project manager:

11. Research Team Contact Details

**Name**	Dr Dinh Ngoc Nguyen
**Position**	Project manager
**Telephone**	+84 37 814 4725

12. Support Services Contact Details

If at any stage you or your child require medical attention, become distressed, or require additional support from someone not involved in the research, please contact your local community health centre.

What if I have a complaint or any concerns about the research study?

If you have any complaints about any aspect of the project, the way it is being conducted, then you may contact the research team in Vietnam, or you may contact the University of New South Wales.

Complaints Contacts

**Position**	Human Research Ethics Office
**Telephone**	+84 262 3825 185
**Reference #**	1804/QĐ-ĐHTN-TCCB

**Position**	Human Research Ethics Coordinator
**Telephone**	+ 61 2 9385 6222
**Email**	humanethics@unsw.edu.au
**Reference #**	HC190136

PARTICIPANT INFORMATION STATEMENT AND CONSENT FORM – Parent Guardian
School and community deworming to control intestinal worm infections
Chief Investigator: Associate Professor Susana Vaz Nery

Consent Form – Parent/Guardian Consent

Declaration by the parent/guardian

- ☐ I understand I am being asked to provide consent for me and my child to participate in this research project;
- ☐ I have read the Participant Information Sheet or someone has read it to me in a language that I understand;
- ☐ I understand the aim of this research and have been given a description of the study tasks and I understand what my child and I will be asked to do;
- ☐ I provide my consent for the information collected about my child and me to be used for the purpose of this research study only.
- ☐ I have had an opportunity to ask questions and I am satisfied with the answers I have received;
- ☐ I understand that I will be given a copy of this document to keep;

I freely agree for me and my child(ren) to participate in this research study as described and I understand that I am free to withdraw at any time, and that withdrawal will not affect my relationship with any of the named organisations and/or research team members.

I agree to participate in filling in a questionnaire/interview YES NO ☐

I agree for my child(ren):

1) ____

2) ____

3) ____

To participate in the following procedures:

- ☐ Provide a stool sample
- ☐ Complete a questionnaire/interview

Parent/Guardian Signature

Signature or thumbprint of parent/guardian	
Date	

Declaration by Researcher/Field worker

- ☐ I have given a verbal explanation of the research study, its study activities and risks and I believe that the participant has understood that explanation.

Researcher Signature*

Name of Researcher (please print)	
Signature of Researcher	
Date	

PARTICIPANT INFORMATION STATEMENT AND CONSENT FORM – Parent Guardian
School and community deworming to control intestinal worm infections
Chief Investigator: Associate Professor Susana Vaz Nery

Form for Withdrawal of Participation

I wish to **WITHDRAW** my consent for my child(ren) to participate in this research study described above and understand that such withdrawal **WILL NOT** affect my relationship with my child's school, teachers, local health centre, The University of New South Wales, or Tay Nguyen University. In withdrawing my consent I would like any information collected from my child that has been provided for the purpose of this research project withdrawn.

Parent/Guardian Signature

Name of Child(ren)<br>(please print)	
Parent/guardian signature	
Date	

The section for Withdrawal of Participation should be forwarded to:

CI Name:	Associate Professor Susana Vaz Nery
Email:	snery@kirby.unsw.edu.au
Phone:	+61 2 9385 0867
Postal Address:	Kirby Institute<br>Wallace Wurth Building<br>High St<br>Kensington<br>NSW 2052<br>Australia
