# Supplementary material for: A cluster-randomised controlled trial comparing school and community-based deworming for soil transmitted helminth control in school-age children: the CoDe-STH trial protocol
Source: BMC Infect Dis. 2019 Sep 18;19:822. doi: 10.1186/s12879-019-4449-6 (PMC6751595; doi:10.1186/s12879-019-4449-6)
Supplement: Supplementary file 2 — Additional file 2. Study questionnaire. [file 12879_2019_4449_MOESM2_ESM.pdf]

STUDENT QUESTIONNAIRE

A. Participant details

1. District (Drop down menu)

2. School (Drop down menu)

3. Participant ID

4. Grade (Drop down menu)

5. Name

B. Health

6. Have you taken deworming drugs in the last year? Y ☐ N ☐ Don't know ☐

C. Sanitation

7. When you are at home and need to defecate, where do you usually defecate? Choose one option.

Household toilet/latrine			
Neighbour toilet /latrine			
Village toilet/latrine			
On the ground/in the bush/grass			
Other		Specify:	

8. Does your house have a toilet/latrine? Y ☐ N ☐ *If no, skip to question 11*

9. Can you flush the toilet with water? Y ☐ N ☐

10. Do you use your household toilet?
    Always ☐
    Sometimes ☐
    Never ☐

11. When you are **at home**, do you ever defecate on the ground? Y ☐ N ☐

12. When you are **at school**, do you ever defecate on the ground? Y ☐ N ☐

D. Personal hygiene

13. Is there a water tap for washing your hands at home? Y ☐ N ☐

	Always	Sometimes	Never
14. Is there soap available to wash your hands at home?	☐	☐	☐
15. Do you wash your hands after defecating?	☐	☐	☐
16. Do you wash your hands before eating?	☐	☐	☐
17. Do you wear shoes when you are outside?	☐	☐	☐
18. Do you wear shoes when going to the toilet?	☐	☐	☐

Thank you for completing the questionnaire.

PARENT/CAREGIVER QUESTIONNAIRE

A. Participant details

1. District: ______

2. School name: ______

3. List the names and grades of your children who attend this school **(in grades 1-4 only)**.

Name	Grade	ID

B. Water

4. What is the main source of DRINKING water for your household? Choose one option.

Piped water to your house or yard	
Public tap	
Protected dug well	
Unprotected well	
Rainwater	
Surface water	
Other, specify:	
Don't know	

C. Education and employment

5. How much school did the child's mother (or primary **female** caregiver) attend? Choose one option.

Never went to school	
Not finished primary school	
Completed primary school	
Not finished secondary school	
Completed secondary school	
Completed professional training	
Not finished university	
Completed university	
Don't know	

6. What is the main job of the main income earner in your household? Choose one option.

Farmer		
Fisherman		
Animal keeper		
Clerk/administration		
Health worker		
Selling at market		
Unemployed		
Other		Specify:
Don’t know		

7. How much income did the household receive over the last year? Choose one option.

Less than 20,000,000 VND	
VND 20,000,000 – 50,000,000	
VND 50,000,000 – 200,000,000	
More than VND 200,000,000	
Don’t know	

This completes the questionnaire. We are grateful for your participation – thank you.
